# Effect of PCM on the Hydration Process of Cement-Based Mixtures: A Novel Thermo-Mechanical Investigation

**DOI:** 10.3390/ma11060871

**Published:** 2018-05-23

**Authors:** Claudia Fabiani, Anna Laura Pisello, Antonella D’Alessandro, Filippo Ubertini, Luisa F. Cabeza, Franco Cotana

**Affiliations:** 1 CIRIAF—Interuniversity Research Center, University of Perugia, Via G. Duranti 63, 06125 Perugia, Italy; fabiani@crbnet.it (C.F.); cotana@crbnet.it (F.C.); 2 Department of Engineering, University of Perugia, Via G. Duranti 93, 06125 Perugia, Italy; 3 Department of Civil and Environmental Engineering, University of Perugia, Via G. Duranti 93, 06125 Perugia, Italy; antonella.dalessandro@unipg.it (A.D.); filippo.ubertini@unipg.it (F.U.); 4 GREiA Research Group, INSPIRES Research Centre, Universitat de Lleida, Pere de Cabrera s/n, 25001 Lleida, Spain; lcabeza@diei.udl.cat

**Keywords:** cement hydration, thermally efficient concretes, cement-based composites, concrete curing, phase change material, mechanical properties, thermal characterization, smart material

## Abstract

The use of Phase Change Material (PCM) for improving building indoor thermal comfort and energy saving has been largely investigated in the literature in recent years, thus confirming PCM’s capability to reduce indoor thermal fluctuation in both summer and winter conditions, according to their melting temperature and operation boundaries. Further to that, the present paper aims at investigating an innovative use of PCM for absorbing heat released by cement during its curing process, which typically contributes to micro-cracking of massive concrete elements, therefore compromising their mechanical performance during their service life. The experiments carried out in this work showed how PCM, even in small quantities (i.e., up to 1% in weight of cement) plays a non-negligible benefit in reducing differential thermal increases between core and surface and therefore mechanical stresses originating from differential thermal expansion, as demonstrated by thermal monitoring of cement-based cubes. Both PCM types analyzed in the study (with melting temperatures at 18 and 25 ${}^{\circ }$C) were properly dispersed in the mix and were shown to be able to reduce the internal temperature of the cement paste by several degrees, i.e., around 5 ${}^{\circ }$C. Additionally, such small amount of PCM produced a reduction of the final density of the composite and an increase of the characteristic compressive strength with respect to the plain recipe.

## 1. Introduction

Hydraulic binders are substances that harden independently and confer cohesion and resistance to a composite system. In the hardening process, which spontaneously takes place at room temperature, these substances chemically react with water and transform the plastic water-binder system into a solid matrix, with the ability to agglomerate other solid materials [1].

It is clear, then, that hydraulic binders, and particularly cement-based ones, play a crucial role in the constitution of concrete, whose production is about one ton per year for every human being on Earth, and can be considered as the most widely used material in the world [2]. From an environmental point of view, this is particularly significant, given that the production of one ton of the most common type of cement, i.e., the Portland one, is associated with the emission of about 600–1000 kg of CO${}_{2}$ in the atmosphere, accounting for at least 5% of man-made CO${}_{2}$ production worldwide [3]. Furthermore, the manufacturing of ordinary Portland cement (OPC) is a highly energivorous process depleting large quantities of precious raw materials and natural resources.

All this considered, in recent years, several research contributions focused on the reduction of the overall environmental impact of cement and consequently concrete, with the aim of producing greener products with comparable mechanical performance. In detail, two different approaches have so far been investigated by the research community. The former one consists of replacing part of the cement with more sustainable components such as waste materials, e.g., wheat straw ash and bamboo leaf ash [4,5], or byproducts from the iron industry or coal combustion, e.g., slag, silica fume and fly ash [6,7,8,9,10]. Fly ash demonstrated being able to improve the overall manufacturing process with the aim of controlling the amount of sensible heat released by the cement during its hydration phase, and reducing the temperature-induced strains and stresses within the solid matrix [11,12]. This last approach allows for obtaining higher strength concretes by using the same amount of resources and would consequently reduce raw material depletion and green house gas emissions imputable to the cement industry.

In order to address this latter application, a better understanding of the kinetics of the hydration mechanism itself is still needed. For this reason, several researchers worldwide investigated the chemical processes occurring during the development of the cement-based solid micro-structure [13,14]. Unfortunately, these chemical processes may operate in parallel, in series or in even more complex combinations. For this reason, for the time being, it is not possible to have a complete picture of the reactions taking place during cement hydration. However, by studying the heat released during the process as a function of the hydration time, it is possible to divide such duration into five distinct stages with specific characteristics, i.e., (i) pre-induction; (ii) induction; (iii) acceleration; (iv) post-acceleration and (v) diffusion-limited reaction [1]. The highest amount of hydration heat is generally released during the acceleration and the post-acceleration phase, which usually take place between two and ten hours from the beginning of curing [15,16].

In the context above, several studies have been investigating the effect of different additives or cement substitutes on the hydration heat evolution. Bouasker et al. for example, focused on the influence of limestone filler and granular inclusions on chemical shrinkage development and on the hydration degree of cementitious matrices. They found that the addition of the milestone filler causes an acceleration of both the hydration process and the chemical shrinkage, while the presence of the granular inclusions with diameters between 1 and 2 mm only has a weak influence on these factors [17]. Hu et al., on the other hand, investigated the effect of slag and fly ashes on cement-based mortars in terms of both hydration heat and early-age chemical and autogenous shrinkage [18]. They observed a linear reduction of the autogenous shrinkage with increasing slag and fly ash replacement levels and also found a good correlation with the amount of heat released during the process.

The relation between hydration heat and shrinkage-induced cracking in the early-age curing of cement-based composites has also been investigated by means of analytical and numerical modeling [19,20,21,22]. Zreiki et al. for example, developed a 3D thermo-chemo-mechanical model in order to assess the risk of cracking in massive concrete structures [19], while Yuan and Wan predicted the early-age cracking of concrete by using a numerical code based on micromechanical model and empirical formulas [20].

In general, all these contributions revealed an inverse correlation between the amount of heat released during the hydration process and the mechanical resistance of the investigated samples. Based on this evidence, in recent years, the attention of the scientific community has been focusing on the implementation of advanced techniques aimed at reducing the release of sensible energy during the chemical transformation of the cement-based composites. In particular, the implementation of a specific kind of latent heat storage material, i.e., phase change material (PCM), within the reacting cementitious matrices is receiving increasing consent among researchers worldwide. Phase change materials are substances capable of storing a large amount of heat in the form of phase change enthalpy, at a constant temperature. Until now, they have mostly been integrated in concrete mixtures with the aim of improving the thermal-energy behavior of a specific building component during the operative phase of the building itself. As a matter of fact, several contributions already testified their potentiality as passive regulator of the thermal fluctuations in the indoor built environment [23,24,25]. However, in the last few years, some contributions have also explored their potential benefit in reducing the amount of heat released during cement hydration [26,27,28,29,30].

In their study about the influence of two different kinds of paraffin-based microencapsulated PCMs on the microstructure and strength development of cementitious composites, Aguayo et al. [29] found a different behaviour of cement mortars and pastes. The former were, in fact, found to behave differently with respect to the specific considered PCM, while the latter decreased their mechanical performance consistently with increasing PCM volume fraction [29]. Fernandes et al. also used paraffin-based microencapsulated phase change materials to produce and mitigate thermal cracking of cement mortars [30]. They found that PCM inclusion did not influence the chemical reactions in the cementing material, but their presence was able to reduce the peak temperature during cement hydration. However, such reduction was relatively low due to dilution, and the final compressive strength of the composites was significantly lower than that of the reference product [30]. A consistent compressive strength reduction with increasing PCM content in concrete mixtures was also found by Cellat et al. [27].

Most of the above referenced studies considered the integration of relatively high PCM volume fractions in the original mix design of the cement-based composites, i.e., from 5% to 20%, which in most cases was shown to cause a redistribution of the void structure and to produce weak interfacial bonds between the PCM and the cement grains. However, as reported in a previous study conducted by this research group, concretes with 1% weight content of PCM with respect to the final weight of the cement/water composite can be associated with a significant reduction in the statistical dispersion of the axial compressive strength with respect to classic concrete [25].

All this considered, this work aims at investigating both the heat storage potential during the hydration phase and the mechanical performance of cement pastes produced by adding 1% in weight of two different paraffin-based phase change materials. The final goal of this study is to produce a thermally enhanced composite capable of improving its mechanical performance by reducing the thermal stresses developed during the early-age hydration phase of the mixture.

## 2. Materials and Methodology

### 2.1. PCM Selection

Two different types of phase change materials were selected as additives for producing the cement pastes investigated in this work, i.e., the Micronal DS 5038X (Microtek, Dayton, OH, USA), and the Microtek mPCM18D micro-capsules (Microtek, Dayton, OH, USA).

A microencapsulated PCM consists of an external inert polymeric shell enclosing the inner latent heat storage medium in order to prevent the leakage phenomenon once the PCM is in its liquid form. Both the selected micro-capsules use paraffin-wax as the core material of the shell; however, the former is associated with a melting temperature of about 25 ${}^{\circ }$C, while the latter of about 18 ${}^{\circ }$C. The PCM content in each capsule is about 85–90 wt % with respect to the whole weight. The capsules look like a white powder with a mean particle size of about 14–24 μm, and 20–300 μm for the mPCM18D and the DS 5038X, respectively; however, the capsules from Micronal laboratories are an agglomeration of smaller shells with a diameter between 1 and 5 μm.

### 2.2. Mix Design and Sample Preparation

The selected PCM microcapsules were used to prepare two different cement pastes with a fixed amount of thermally enhanced additive, i.e., 1% with respect to the total cement weight. A standard cement paste without the addition of PCM was also prepared for comparative purposes (NP). Therefore, three different kinds of pastes were considered: a normal cement paste (NP), a paste additivated with 1% of microencapsulated phase change material with melting point at 18 ${}^{\circ }$C (PCM18P), and a paste additivated with 1% of microencapsulated phase change material with melting point at 25 ${}^{\circ }$C (PCM25P). The research focus was dedicated to the fundamental cement curing process. Therefore, no aggregates were considered because they might also affect the curing process and interact with PCM. In the PCM-doped composites, the additives were used in addition to the reference mix design, in order for them to have the same water/cement ratio of the original product and, consequently, comparable mechanical results. The three different mix designs are summarized in Table 1.

**Figure 1:**
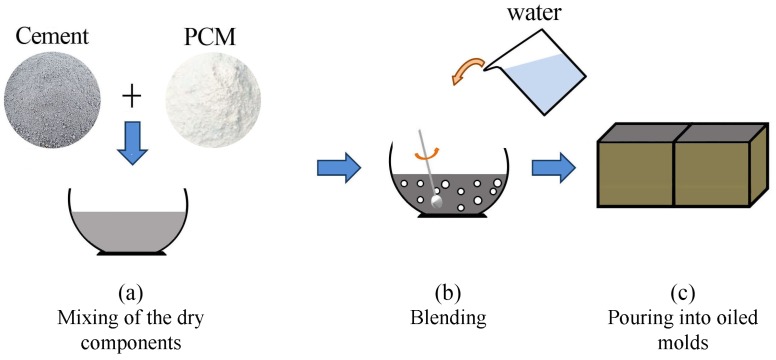
Samples preparation procedure: (**a**) assembly of the dry components; (**b**) addition of water and mixing; and (**c**) pouring into oiled molds.

Fifteen cubic samples were produced in total, i.e., five samples per mix design, with a dimension of 10 × 10 × 10 cm${}^{3}$. The preparation of the five cement pastes was carried out by following the same procedure (Figure 1). First of all, the dry materials (cement and PCMs) were carefully manually merged in order to obtain an homogeneous powder mix. Consecutively, water was added to the compound, which was then carefully blended trying to preserve the capsules from brittle fractures due to the mechanical action. Finally, after reaching the required workability, the mixtures were poured into previously oiled molds and compacted in order to improve homogeneity and eliminate voids and air inclusions. Before the moulding of the samples, the workability was investigated through a modified Abram’s cone test. The comparable and acceptable results avoided the use of any types of fluidifying additives. Finally, the samples were cured within the controlled environment of an empty, fully monitored test building, which was kept at the temperature of 16 ± 0.5 ${}^{\circ }$C by using a built-in conditioning system during the whole curing process (28 days). After seven days, the specimens were unmolded within the same controlled environment where they were housed for the rest of the curing process.

## 3. Thermo-Mechanical Analyses

Thermo-mechanical behaviour of the samples was analysed by means of a novel dynamic thermal procedure and acknowledged mechanical tests. From the thermal point of view, two different investigations were carried out with the aim of (i) monitoring the temperature evolution of the cementitious matrices during the hydration process and (ii) investigating the dispersion of the PCM characterizing the morphological features of the composite. From a mechanical point of view, both elastic modulus and compressive strength were evaluated. In addition, workability of the volume, weight, and density were measured.

### 3.1. Thermal Characterization of the PCMs

The thermal behaviour of both the microcapsules alone was analysed by means of the transient plane source (TPS) method at two different constant temperatures, i.e., 10 ${}^{\circ }$C and 35 ${}^{\circ }$C. The capsules were placed in a specifically designed sample holder, equipped with a TPS sensor, and housed within the controlled environment of a climatic chamber. They were kept at the selected temperature for 36 h and five subsequent measurements were carried out (1 every 60 min), during the last 4 h of the thermal stabilization.

**Figure 2:**
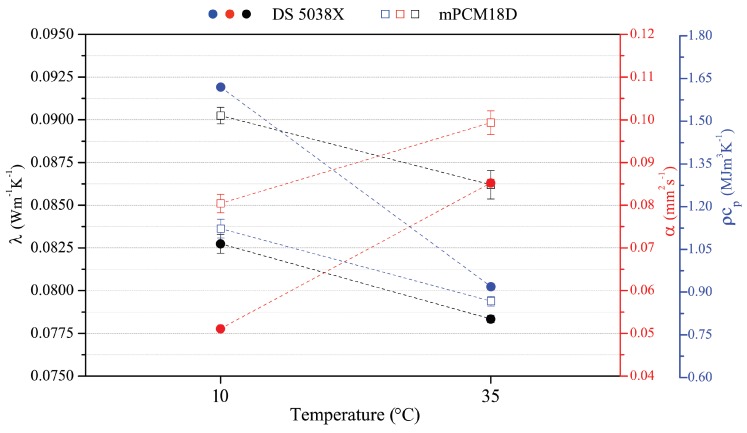
Thermal conductivity, thermal diffusivity and the volumetric specific heat of the DS 5038X and the mPCM18D microcapsules at 10 and 35 ${}^{\circ }$C.

Figure 2 shows the thermal conductivity, thermal diffusivity and volumetric specific heat of the PCM. By comparing the results achieved in both solid and liquid PCM configurations, different performance rates were achieved, e.g., both the capsules decrease their thermal conductivity and their volumetric specific heat with varying the test temperature from 10 ${}^{\circ }$C to 35 ${}^{\circ }$C, while their thermal diffusivity increases. Results show that the DS 5038X PCM is associated with lower thermal conductivity and diffusivity, while it possesses higher volumetric specific heat, if compared to the mPCM18D. The whole PCM powder made by microcapsules was characterized by means of such method, since the microcapsules are radically smaller than the TPS probe detection capability specifically selected for this purpose. The TPS probe and method selection were driven by the purpose to characterize the whole hemispherical volume detected by the selected probe, whose diameter is of the order of magnitude of centimeters, i.e., 1.4 cm. This selection was specifically carried out with the purpose to characterize the thermal properties of the whole powder itself, as typically done for any other additives responsible for thermal modification of the composite materials. Since both the selected PCM microspheres represent already commercial products with available technical sheets and proper documentation, the reader is kindly referred to [31,32] for further details such as henthalpy of transition phases. As previously mentioned, the whole microcapsules quantity was characterized at stabilized temperature, without interacting with the PCM transition phase itself. In this view, the TPS analyses refer to the whole volume occupied by the microcapsule, where the TPS is able to detect the hemispherical volume under its influence. This procedure may therefore produce non-consistent results compared to the ones about the PCM paraffin itself, but it has been considered as significant for investigating the role of such microcapsule volume included into the cement paste.

### 3.2. Rheologial Behaviour of the Pastes

As soon as the cement-based compound was produced, the fresh cement paste workability was investigated through a flow test carried out with a hollow truncated cone with a height of 90 mm and maximum and minimum diameters of 70 mm and 40 mm, respectively. The value related to the workability was obtained by evaluating the average of orthogonal diameters of the flow on a livelled metallic table after the mold was removed. The results show very similar performance, index of similar consistency of the mix designs. In particular, NP, PCM18P and PCM25P demonstrated average diameters of 18.5, 18.2 and 18.3 mm, respectively. This is particularly interesting, also considering that, as reported in Section 2.2, no plasticizer was added to any of the different mixes.

### 3.3. Thermal Monitoring of the Hydration Process

**Figure 3:**
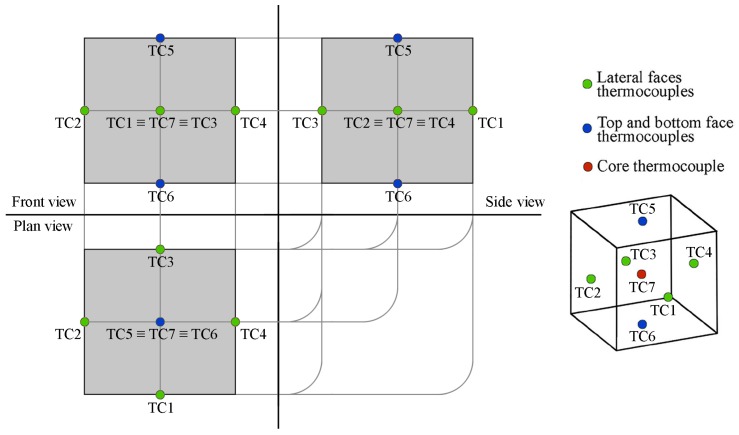
Thermocouples positioning within the cementitious samples.

The thermal monitoring of the samples during the hydration process was carried out by using seven T-type thermocouples for each mix design. In more detail, six of these sensors were placed at the center of each samples’ face while the last one was introduced within the main core of the specimen. In this way, it was possible to accurately register the temperature gradients produced throughout the whole volume of the samples during the overall extent of the hydration process (Figure 3).

The thermal analysis of the cement pastes was carried out inside polyurethane molds, with the same boundary conditions. This configuration was selected because, in cement-based materials, the critical exothermic reaction takes place in the first hours after casting and because insulating material that constitutes the formwork simulates the occurrence of more massive pourings. The thermocouples were connected to a data acquisition system model cDAQ-9184 equipped with two NI 9213 Spring slots (National Instruments, Austin, TX, USA), and programmed in order to read the sensors every 60 s during the next 28 days.

The samples were housed within the controlled environment of a test-building, where a conditioning system was used to keep the ambient temperature at 16 ± 0.5 ${}^{\circ }$C. This particular temperature was chosen in order to allow both the selected PCM to eventually turn from solid to liquid and vice-versa due to the hydration heat released by the cementitious matrix.

### 3.4. Thermal Behaviour and PCM Distribution Analysis

As previously described in Section 2, the basic thermal properties of the PCM used as additive for the production of the composite cement pastes were firstly characterized by using the transient plane source (TPS) technique. This thermal analysis was carried by using a Hot Disk 2500S apparatus (Hot Disk AB, Göteborg, Sweden), which was also used to investigate the proper PCM dispersion in the final mixture, by means of a non-destructive experimental procedure.

The Hot Disk 2500S is an experimental piece of equipment able to implement a transient heat source to investigate the bulk thermal properties of a material [33,34]. In the general procedure, a certain heating power *P*${}_{0}$ is provided by a double nickel spiral to the sample for a specific amount of time *t*. During the measurement, the resistance of the probe *R*(*t*) is continuously monitored and finally related to thermal properties of the bulk material by using Equation (1):

(1) $$R\left(t\right)=R_{0}\left[1+a\left(\Delta T_{i}+\left(\frac{P_{0}}{\pi^{3/2}r\lambda }\right)D\left(\tau \right)\right)\right],$$

where *t* is the test time [s], $R_{0}$ is the initial resistance of the sensor at time *t* = 0 [Ω], *a* is the temperature coefficient of resistivity (TCR) [1/K], $\Delta T_{i}$ is the temperature difference across the insulating layers of the probe [K], *D*(τ) is the dimensionless shape function and τ is the dimensionless time, which is a function of sample thermal diffusivity (α).

Besides the classic bulk analysis, the TPS method has recently been applied for investigating the variation of thermal conductivity as a function of the probing depth in inhomogeneous materials [35]. As a matter of fact, in the TPS method, the thermal penetration depth can be determined as a function of the measurement time by means of Equation (2):

(2) $$d_{p}=2\sqrt{\alpha t},$$

where *d*${}_{p}$ represents the experimental probing depth.

Equation (2) can be used to extend the mathematical model and approximate the thermal conductivity across the overall thickness of inhomogeneous materials. In order to do so, the fundamental fitting procedure of the TPS method is applied to smaller time windows, [*t*${}_{i}$, *t*${}_{i}+n\Delta t$], where *n* is the number of points in the time window itself, and Δt is the sampling time step, producing locally averaged thermal conductivity and diffusivity values. In this way, by sliding the selected time window across the entire measurement time range, different thermal conductivity values are estimated along the probing depth of the sample.

**Figure 4:**
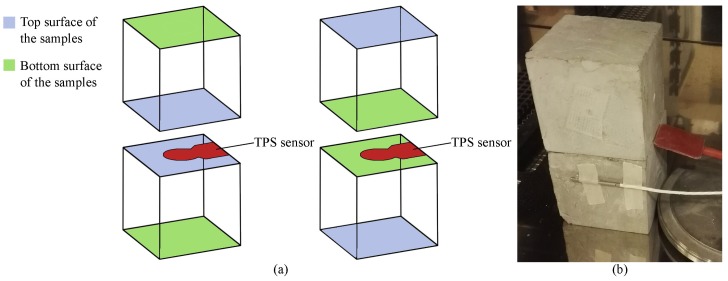
(**a**) schematic of the TPS (transient plane source) sensor and sample positioning; and (**b**) final experimental setup during the structural analyses using the Hot Disk 2500S.

In this work, both these analyses were used to define the average thermal properties of the considered mixtures and also indirectly identify the dispersion of the phase change material in the cement paste. This last application was possible thanks to (i) the huge contrast between the thermal properties of the cementitious matrix and the PCMs; and (ii) the highly different weight percentage of the two components (see Table 1). In detail, once the samples completed their curing process, they were placed within the controlled environment of a climatic chamber and kept at the fixed temperature of 10 ${}^{\circ }$C. Once the pastes reached a stationary temperature profile, they were analysed by means of the bulk and the structural double-sided module of the TPS method. The sensor was sandwiched between two identical samples of the same kind, the first time in direct contact with the two top surfaces of the samples, and the second time in direct contact with the bottom surfaces (see Figure 4). In this way, the thermal conductivity evolution was analysed through the overall thickness of the different samples. The same procedure was later repeated by conditioning the samples at 35 ${}^{\circ }$C.

### 3.5. Physico-Chemical and Mechanical Analysis

**Figure 5:**
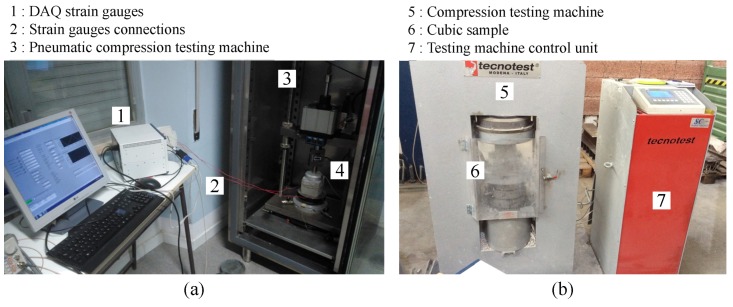
Experimental setup of the (**a**) cyclical compressive tests and (**b**) compressive tests up to break.

After curing, all the samples were measured, weighed and observed in order to identify specific peculiarities and any defects. The elastic moduli were evaluated through cyclical compressive loads between 0.5 and 9 kN applied with a speed of 1 kN/s using a servo-controlled pneumatic universal dynamic testing machine, model IPC Global UTM14P (IPC Global Pty Ltd., Boronia, Australia) with a controlled temperature chamber. Three samples of each type of paste were instrumented with two 2-cm long strain gauges placed on lateral opposite sides having a nominal resistance of 120 Ω and Gauge Factor of 2. Compression tests up to the maximum strength were carried out using a Tecnotest compressive machine (Tecnotest, Marghera, Italy) with load control, according to UNI EN 12390-3 (UNI EN 1239-3 “Testing hardened concrete—Part 3: Compressive strength of test specimens” May 2009). The nominal speed was 5 kN/s. The setup of the cyclical and ultimate compressive tests are reported in Figure 5.

Average and characteristic compressive strength were calculated from the results. The characteristic values were obtained using the formula *R*${}_{ck}$ = *R*${}_{m}$ − *k*σ, where *R*${}_{m}$ is the average strength, σ the standard deviation of the compressive strength values of the tested samples, and *k* is a coefficient depending on the number of tested samples, assumed equal to 3.4 [36]. The characteristic strength can be equivalently expressed as: $R_{CM}=R_{m}\left(1-kCOV\right)$, where COV is the coefficient of variation, i.e., the ratio between the standard deviation and the average strength.

## 4. Results and Discussion

### 4.1. Thermal Characterization of the Hydration Process

**Figure 6:**
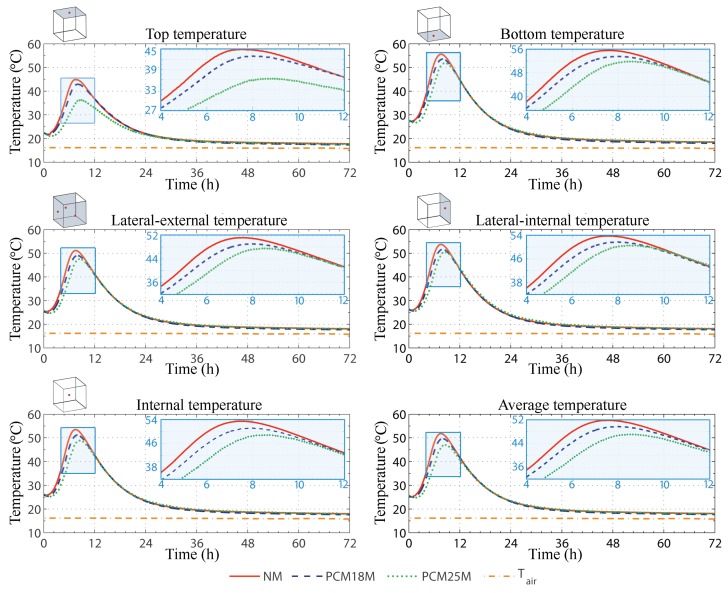
Monitored temperature waveforms of the NP (normal cement paste), the PCM18P (paste with mPCM18D) and the PCM25P (paste with DS 5038X) samples.

Results from the thermal monitoring of the first 72 h of the hydration process are shown in Figure 6, where the dot-dashed line represents the environmental forcing imposed by the conditioning system on the controlled air domain surrounding the samples.

Every monitored temperature profile stabilizes at about 16 ${}^{\circ }$C after the considered time frame, which fully embraces the exoenergetic reaction between cement and water; for this reason, here, only the temperature trends of the first three days are shown.

The graphs in Figure 6 display the temperature trend registered by the different thermocouples for each of the three kinds of pastes, i.e., the NP, the PCM18P and the PCM25P sample. The three lateral thermocouples directly exposed to the conditioned environment are reported here by considering their average value, while the fourth one, i.e., the one insisting on the side of the mold, which was in direct contact with another sample of the same kind, is separately reported.

In general, Figure 6 shows a single temperature peak for every investigated sample. Such peak can be associated with the acceleration and the post acceleration period of the hydration process, since the actual monitoring of the samples started after 40 min after adding water to cement and PCM. By looking at the different temperature waveforms, the NP sample is always associated with the highest temperature values, always reaching peaks above 50 ${}^{\circ }$C, with the exception of the top surface, which is clearly associated with the highest heat flux through the local controlled environment. The PCM25P is the sample with the lowest amount of sensible heat released during the hydration process, while the PCM18P shows an intermediate trend, if compared with the two previous pastes.

It is noteworthy that the highest temperatures are always registered at the bottom and at the lateral internal surface of the samples and not in the main core of the cement pastes. This is probably due to two different factors: first of all, both these sensors are placed within the center of a cube face that is not directly exposed to the conditioned environment, secondly, the molds are made by a 20-mm thick PUR layer, which is a plastic material characterized by a fairly low thermal conductivity. As a consequence, these two thermocouples are in direct contact with a volume of cement, which cannot easily release heat, and is forced to store a higher amount of sensible energy in its matrix.

**Figure 7:**
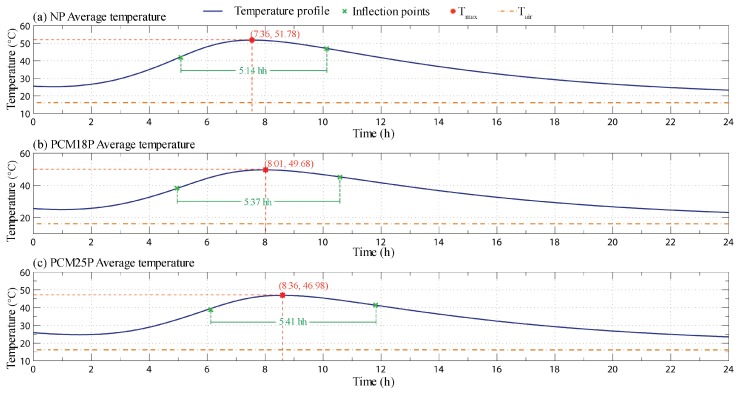
Average monitored temperature profiles for the first 24 h of the hydration process for (**a**) the NP; (**b**) the PCM18P and (**c**) the PCM25P sample, with the respective maximum and inflection points.

By taking a closer look to the average profiles defined for every analysed cement paste, it is possible to have a more detailed idea of the final effect that even a small amount of phase change materials can produce during the hydration reaction when they are used as additives for the production of thermally enhanced cement-based composite (see Figure 7).

The main temperature peak associated with the system reduces from 51.8 ${}^{\circ }$C in the NP sample to about 49.7 ${}^{\circ }$C and 47.0 ${}^{\circ }$C for the PCM18P and the PCM25P sample, respectively. From these data, the use of the PCM25P shows a higher effect on the actual reduction of the peak temperature registered during the observed exoenergetic reaction taking place in the mixture. This is probably due to the influence of the pre-induction and induction periods of the hydration process. At the beginning of the monitored period, in fact, all of the samples are associated with temperature values around 25 ${}^{\circ }$C, thus well above the melting point of the mPCM18D, and, because of this, no phase transition could happen in the PCM18P paste at this stage of the curing.

However, the lower thermal conductivity and diffusivity of the paraffin microcapsules compared to those of the cementitious matrix (see Section 4.2) allows for releasing the heat produced during the hydration process at a slower rate. This slightly reduces the maximum peak temperature registered by the sensors, while producing a wider thermal wave. As a matter of fact, the time interval separating the two inflection points of the thermal waves increases of about 23 min with the addition of the mPCM18D microcapsules, i.e., from 5 h and 14 min to 5 h and 37 min.

The same effect can also be seen when the temperature profile of the PCM25P paste is considered. However, in this case, the effect of the phase change also produces a time lag in the temperature trend. As it can be seen in Figure 7, the peak value of the monitored profile in this case is only reached after 8 h and 36 min, which is about 1 h later than the NP paste and 35 min later than the PCM18P paste.

### 4.2. Thermal Characterization and PCM Distribution Results

As previously described in Section 3.4, in this work, the transient plane source method was used to define the basic thermal properties of the cured samples at two different temperatures, i.e., 10 and 35 ${}^{\circ }$C, and also to investigate the actual PCM distribution in the final product. Table 2 shows the average values of thermal conductivity, thermal diffusivity and volumetric specific heat obtained using the classic bulk TPS analysis on the different samples. As can be seen, the introduction of the PCM in the cement-based mixture does not significantly influence the final thermal behaviour of the samples. However, every additivated paste is generally associated with lower volumetric specific heat and thermal diffusivity, and higher thermal conductivity. Additionally, the TPS denotes similar differences among comparable measurements carried out at different temperature conditions, particularly in the case of the PCM-doped recipes, i.e., PCM18P and PCM25P. As a matter of fact, the additivated samples always reduce their thermal conductivity and diffusivity with increasing temperature of the material, while their volumetric specific heat generally slightly increases.

**Figure 8:**
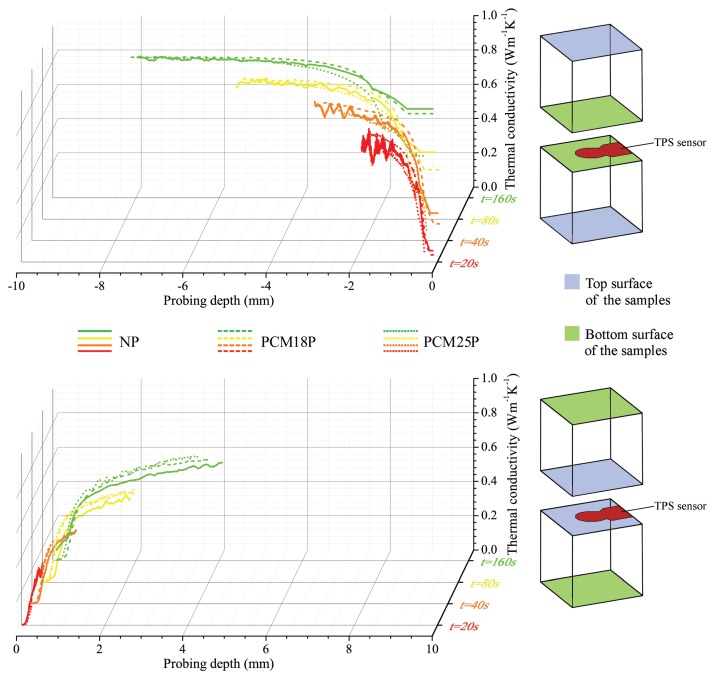
Thermal conductivity profiles as a function of the probing depth for the three different samples (NP, 18PCMP and 25PCMP) at 35 ${}^{\circ }$C, considering four different probing times: 20, 40, 80 and 160 s.

As regarding the PCM distribution analysis, also in this case, the characterization procedure was repeated at 10 and 35 ${}^{\circ }$C, however, and, as expected, no significant differences can be noticed in the two cases. For this reason, only the results at 35 ${}^{\circ }$C are directly reported in Figure 8, which shows the obtained thermal conductivity trends at a maximum distance of about 8 mm through the thickness of the cement-based pastes.

All the samples present similar profiles, which can be considered as a first indication about the relatively good dispersion of the PCMs in the mixtures. Smaller measurement times are always associated with a noisier signal which typically characterizes the portion of the pastes directly adjacent to the investigated surface, but can only penetrate short probing depths. With increasing measurement times, on the other hand, the thermal wave diffuses more deeply into the samples, thus the 2000 measurement points taken by the Hot Disk during the considered transient produce less accurate values in the superficial volume of the cubes, but allow for more deeply investigating the thermal conductivity evolution.

By comparing the thermal conductivity trends through the bottom surface of the different cement-based pastes, a huge and abrupt increase is generally found, and the thermal conductivity of the deepest points very closely approximates the average values obtained with the bulk analysis and listed in Table 2, for each of the considered specimens. Less sloped curves, typical of materials with higher insulation capability, on the other hand, characterize the signal recorded through the top surface of the pastes. Additionally, in this case, even using the largest measurement time, i.e., 160 s, the obtained λ value always remains well below the average results in Table 2. Such peculiar behaviour is detected in all the samples, and is even more pronounced when the normal paste is considered, so it can be safely stated that it is not an effect produced by a differential dispersion of the latent additive. On the contrary, this peculiar trend is most probably due to two different phenomena taking place simultaneously. The first one can be considered as an instrumental error, in fact, by sandwiching the TPS sensor between the two upper surfaces of the cement cubes, a higher contact resistance was detected by the double spiral. This is due to fact that the top-free surfaces of the samples are in direct contact with air during the overall duration of the curing process, and, for this reason, they develop a more irregular plane, with a higher number of voids, and, consequently, higher surface resistance. The second phenomenon can be associated with a sort of differential sedimentation occurring in the pure cement paste, which causes less dense components, particularly the air trapped in the cement paste during the blending procedure, to float in the mixture, and be mostly accumulated in the upper part of the sample.

In general, the NP, PCM18P and PCM25P curves are similar; however, the bottom surface profiles show that (i) the NP sample produces the highest thermal noise; (ii) the PCM18P thermal conductivity trend is always the highest one; and (iii) the PCM25P always presents the lowest thermal conductivity. This is not true for λ profiles obtained by interposing the TPS sensor between the top surfaces of the cement cubes. In this case, as a matter of fact, the normal paste (NP) is always associated with the lowest thermal conductivity trend.

### 4.3. Physico-Thermal and Mechanical Characterization

**Figure 9:**
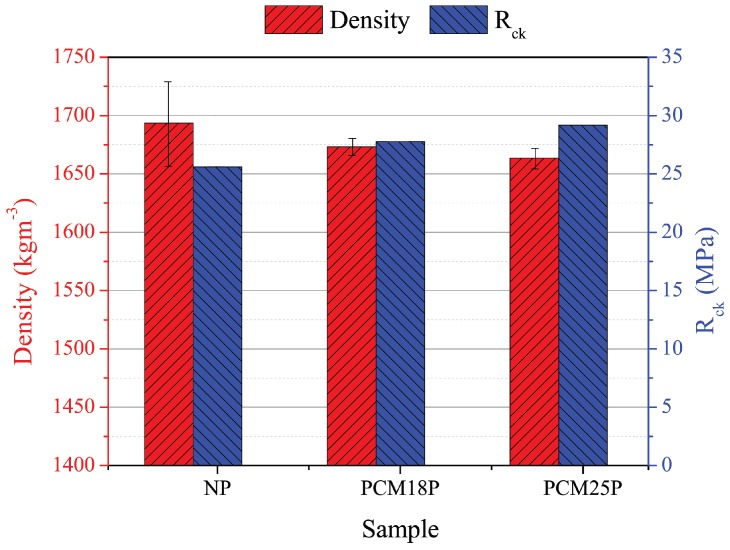
Average density and characteristic strength of the NP, PCM18P and PCM25P samples.

The analysis of the average density showed, as expected, minor values for mixes with PCM, expecially in the case of PCM25P. In addition, the coefficients of variation of doped cement pastes were relatively lower compared to normal pastes: 0.005 and 0.007 for PCM18P and PCM25P, respectively, against 0.027 of NP. Table 3 shows the physical characteristics of the 15 samples, i.e., the values of volume, weight, density and their standard deviation and coefficient of variation. Figure 9 shows the variation of density and its standard deviation of the three different cement-paste materials, together with their characteristic strength. Table 4 shows the elastic moduli obtained from cyclical loading tests together with the values of the average strain measured through the strain gauges. The results demonstrate that the PCM presence decreases the elastic moduli of the cementitious materials, especially in the case of PCM18P, which exhibits a Young modulus E = 8784 MPa, about one half the Young modulus of normal cement paste. The results of strength compressive tests are reported in Table 5. The table shows the results of all 15 samples, in terms of maximum force and resistance. Average compressive strength was calculated for each type of cement paste. The analysis was carried out without considering the first NP sample and the last PCM25P sample, which were clear outliers if compared to the others. The average strength of the normal samples was 34.94 MPa while PCM18P and PCM25P exhibited values of 37.24 and 37.52 MPa, respectively, about 7% higher than the NP.

### 4.4. Discussion of the Results

Results from the thermo-mechanical characterization of the different cement pastes produced in this work can be used to draw some interesting conclusions about the use phase change materials within cement-based matrices. As a matter of fact, the thermal analyses carried out on the samples showed that the introduction of a small amount of PCM, i.e., 1% in weight of cement, can effectively reduce the peak temperature reached by the composites during the first 24 h of the curing process. Because of this, the PCM-doped matrices are exposed to lower temperature gradients throughout their volume and, consequently, to a more uniform thermal expansion, which, in turn, reduces the amount of strains and stresses that inevitably are generated in the solid volume as a consequence of the exoenergetic hydration reaction.

The non-destructive PCM-distribution analysis carried by using the TPS method, on the other hand, allowed for concluding that, despite the cement paste itself exhibiting an intrinsic dis-homogeneity, probably due to a small amount of differential sedimentation driven by density gradients in the paste, the latent additives were mostly well mixed during the production phase of the samples.

The promising thermal results were confirmed by the mechanical characterization of the samples in terms of density, and characteristic strength, although a notable reduction in Young moduli was made evident due to soft PCM inclusions. In particular, the introduction of 1% in weight of phase change material was able to increase its characteristic compressive strength by about 7% for both samples, while also reducing the mass density of the final composite. Characteristic compressive strength and COV are important values for structural applications: the first represents the strength that 5% of samples do not reach, while the COV is a measure of the reliability of the structural material. Both doped cement pastes exhibit higher characteristic strength and lower COV, which demonstrate a good structural performance. In particular, NP, PCM18P and PCM25P showed Rck and COV of 25.60, 27.77, 29.18 MPa and 0.079, 0.075 and 0.065, respectively.

## 5. Conclusions

In this work, a small amount of micro-capsulated PCM, i.e., 1% in weight, is introduced in a cement paste with the aim of improving its mechanical performance by reducing the sensible heat released during the hydration process and, consequently, the micro-cracking developed because of the thermally induced differential dilatation of the solid matrix. Two different paraffin-based latent additives are considered and compared in this study, i.e., the Micronal DS 5038X and the Microtek mPCM18D micro-capsules. These additives present similar thermophysical characteristics, but they have two different melting temperatures, i.e., 18 and 25 ${}^{\circ }$C.

The thermal monitoring during the 28 days of curing showed how the latent nature of the PCM plays a significant role in reducing the sensible heat released during the first 24 h of the hydration process. In particular, the peak temperature registered for the standard cement paste was lowered by about 4 ${}^{\circ }$C due to the introduction of the micro-capsulated PCM with a phase change temperature of 18 ${}^{\circ }$C, and by about 5 ${}^{\circ }$C if the micro-capsulated PCM with a 25 ${}^{\circ }$C threshold is taken into account. The latent additives were also shown to positively affect the dynamics of the heating process. In fact, the obtained temperature peaks were delayed by about 35 and 60 min thanks to the addition of the Microtek mPCM18D and the Micronal DS 5038X micro-capsules, respectively. Lastly, the time distance between the inflection points characterizing the monitored thermal waves increases from 5 h and 14 min to 5 h and 37 min and 5 h and 41 min if the micro-capsulated PCM with a melting temperature of 18 ${}^{\circ }$C and the latent additives with a melting temperature of 25 ${}^{\circ }$C are considered, respectively.

The positive thermal effect of the PCM was also corroborated by the mechanical analyses carried on the samples. In particular, the PCM-doped pastes increased their characteristic compressive strength of about 7%, despite the non-negligible reduction in density in the final composite, reaching an average characteristic strength of 27.77, and 29.18 MPa for the PCM18P and PCM25P sample, respectively, against the obtained value of 25.60 MPa of the NP one. Additionally, the introduction of 1% in weight of PCM also allowed for obtaining lower coefficients of variation (COV), i.e., 0.079, 0.075 and 0.065, for the NP, the PCM18P and PCM25P paste, respectively. According to the thermal analyses, cement pastes with PCM with a melting point at 25 ${}^{\circ }$C showed the best performance in terms of both resistance and reliability.

In conclusion, the thermo-mechanical investigations carried out in this work demonstrated the positive effect deriving from the introduction of a small amount of PCM, i.e., 1% of the total cement weight, in common cement–water composites. This innovative solution could represent an effective way to improve the final mechanical performance of concrete castings subjected to extensive thermal expansion phenomena, such as massive castings and castings at high temperatures.

## Figures and Tables

**Table 1:** Mix designs of the produced cement pastes, i.e., normal paste (NP), paste doped with microencapsulated phase change material with melting point at 18 ${}^{\circ }$C (PCM18P), and doped with microencapsulated PCM with melting point at 25 ${}^{\circ }$C (PCM25P).

Components	NP	PCM18P	PCM25P
Cement (g)	1277	1277	1277
Water (g)	575	575	575
PCM (g)	-	12.77	12.77
*w*/*c* ratio	0.45	0.45	0.45

**Table 2:** Thermal conductivity (λ), thermal diffusivity (α) and volumetric specific heat (ρc${}_{p}$), with the relative standard deviation, i.e., $\sigma_{\lambda }$, $\sigma_{\alpha }$ and $\sigma_{\rho c_{p}}$, of three considered samples (NP, PCM18P, PCM25P), at different temperature (T) conditions.

Samples	T	λ	$\sigma_{\lambda }$	α	$\sigma_{\lambda }$	ρc${}_{p}$	$\sigma_{\rho c_{p}}$
	(${}^{\circ }$C)	$\left(\frac{W}{\mathrm{mK}}\right)$	$\left(\frac{W}{\mathrm{mK}}\right)$	$\left(\frac{{\mathrm{mm}}^{2}}{s}\right)$	$\left(\frac{{\mathrm{mm}}^{2}}{s}\right)$	$\left(\frac{\mathrm{MJ}}{m^{3}K}\right)$	$\left(\frac{\mathrm{MJ}}{m^{3}K}\right)$
NP	10	0.794	0.001	0.378	0.001	2.102	0.019
	35	0.791	0.004	0.371	0.009	2.134	0.045
PCM18P	10	0.812	0.003	0.370	0.008	2.197	0.050
	35	0.810	0.003	0.368	0.004	2.204	0.016
PCM25P	10	0.813	0.011	0.382	0.147	2.131	0.063
	35	0.763	0.011	0.354	0.014	2.156	0.059

**Table 3:** Measured volume (*V*), weight (*w*), density (ρ), average weight ($\bar{w}$), average density ($\bar{\rho }$) and relative standard deviation (σ) and coefficient of variance (COV) of the 5 NP, PCM18P and PCM25P samples.

	Sample	*V* (mm${}^{3}$)	*w* (g)	$\bar{w}$ (g)	$\sigma_{w}$	COV${}_{w}$	ρ (kg/m${}^{3}$)	$\bar{\rho }$ (kg/m${}^{3}$)	$\sigma_{\rho }$	COV${}_{\rho }$
NP	1	9.9091 × $10^{5}$	1650				1665			
	2	9.8049 × $10^{5}$	1648				1681			
	3	9.9570 × $10^{5}$	1627	1672	41	0.024	1634	1694	46	0.027
	4	9.8288 × $10^{5}$	1705				1735			
	5	9.9013 × $10^{5}$	1708				1725			
PCM18P	1	9.8675 × $10^{5}$	1647				1669			
	2	9.9252 × $10^{5}$	1671				1684			
	3	9.9850 × $10^{5}$	1680	1656.4	19	0.011	1683	1673	9	0.005
	4	9.9113 × $10^{5}$	1649				1664			
	5	9.8067 × $10^{5}$	1635				1667			
PCM25P	1	9.8586 × $10^{5}$	1625				1648			
	2	9.9431 × $10^{5}$	1648				1657			
	3	9.7761 × $10^{5}$	1634	1633.8	9	0.006	1671	1664	12	0.007
	5	9.7967 × $10^{5}$	1627				1661			

**Table 4:** Compressive load variation (ΔF), average sample surface (A), average strain (Δϵ) and average elastic moduli (E) of the NP, the PCM18P and the PCM25C sample.

Sample	ΔF (kN)	A (mm${}^{2}$)	Δϵ	E (MPa)
NP	8.5	9949	4.866 × $10^{-5}$	17,558
PCM18P	8.5	9945	9.730 × $10^{-5}$	8784
PCM25P	8.5	9940	8.106 × $10^{-5}$	10,549

**Table 5:** Peak load (PL), strength (R), average strength ($R_{m}$) and standard deviation ($\sigma_{R_{m}}$), characteristic strength ($R_{ck}$) and covariance (Cv) of the 5 NP, PCM18P and PCM25P samples.

	Sample	PL (kN)	R (MPa)	$R_{m}$ (MPa)	$\sigma_{R_{m}}$	$R_{\mathrm{ck}}$ (MPa)	Cv
NP	1	513.6	51.42				
	2	322.2	32.43				
	3	340.2	34.19	34.94	2.7	25.60	0.079
	4	340.8	34.29				
	5	386.6	38.86				
PCM18P	1	397.6	39.96				
	2	396.3	39.85				
	3	336.1	33.73	37.24	2.8	27.77	0.075
	4	348.8	35.13				
	5	373.7	37.53				
PCM25P	1	382.1	38.44			
	2	375.7	37.78				
	3	395.6	39.79	37.52	2.5	29.18	0.065
	4	338.3	34.06				
	5	302.8	30.41				
